# 2289. Accuracy of the National Early Warning Score 2 (NEWS2) in predicting Mortality and ICU Admission among COVID-19 patients in the Emergency Department of a Tertiary Private Hospital in the Philippines

**DOI:** 10.1093/ofid/ofad500.1911

**Published:** 2023-11-27

**Authors:** Bernice Ong-dela Cruz, Darlene Mae E Cledera

**Affiliations:** Chinese General Hospital and Medical Center, Manila, National Capital Region, Philippines; Chinese General Hospital and Medical Center, Manila, National Capital Region, Philippines

## Abstract

**Background:**

The objective of this study is to evaluate the prognostic value of NEWS2, assessed upon arrival at the emergency department, for predicting in-hospital mortality and ICU admission in COVID-19 patients.

**Methods:**

In this retrospective cohort study, the medical records of patients with confirmed COVID-19 infection between June 2021 to November 2021 were reviewed. The study evaluated the predictive accuracy of NEWS2 for ICU admission and in-hospital mortality based on the patients' baseline scores at the emergency department. Sensitivity, specificity, and the area under the curve were calculated for NEWS2 score of ≥ 5.

**Results:**

A total of 382 COVID-19 patients were analyzed. The median age was 63 years, of whom 59% were male. The overall mortality rate was 23.5%, and 28.5% of patients were admitted to the ICU. The median NEWS2 score was 7 with more than half of the patients considered high risk.The sensitivity and specificity of a NEWS2 score of 5 or higher in predicting ICU admission were 93.58% and 40.66%, respectively, with an area under the curve of 0.67 (95% CI: 0.63-0.71). The sensitivity and specificity of a NEWS2 score of 5 or higher in predicting mortality were 87.78% and 36.64%, respectively, with an area under the curve of 0.62 (95% CI: 0.58-0.67). Age, respiratory rate, oxygen saturation, hypertension, diabetes mellitus, and cardiac disease were found to be associated with an increased risk of ICU admission and mortality.

**Conclusion:**

NEWS2 has good sensitivity and is a reliable predictor of ICU admission and in-hospital mortality in COVID-19 patients

**Disclosures:**

**All Authors**: No reported disclosures

